# Systems genetics analyses predict a transcription role for P2P-R: Molecular confirmation that P2P-R is a transcriptional co-repressor

**DOI:** 10.1186/1752-0509-4-14

**Published:** 2010-02-25

**Authors:** Philippos Peidis, Thomas Giannakouros, Matthew E Burow, Robert W Williams, Robert E Scott

**Affiliations:** 1 Laboratory of Biochemistry, Department of Chemistry, The Aristotle University, 54124 Thessaloniki, Greece; 2 Section of Hematology and Medical Oncology, Tulane University School of Medicine, New Orleans, LA 70112, USA; 3 Varigenix, Inc, Memphis, TN 38120, USA; 4 Lady Davis Institute for Medical Research, McGill University, Sir Mortimer B Davis-Jewish General Hospital, Montreal, Quebec H3T 1E2, Canada

## Abstract

**Background:**

The 250 kDa P2P-R protein (also known as PACT and Rbbp6) was cloned over a decade ago and was found to bind both the p53 and Rb1 tumor suppressor proteins. In addition, P2P-R has been associated with multiple biological functions, such as mitosis, mRNA processing, translation and ubiquitination. In the current studies, the online GeneNetwork system was employed to further probe P2P-R biological functions. Molecular studies were then performed to confirm the GeneNetwork evaluations.

**Results:**

GeneNetwork and associated gene ontology links were used to investigate the coexpression of P2P-R with distinct functional sets of genes in an adipocyte genetic reference panel of HXB/BXH recombinant strains of rats and an eye genetic reference panel of BXD recombinant inbred strains of mice. The results establish that biological networks of 75 and 135 transcription-associated gene products that include P2P-R are co-expressed in a genetically-defined manner in rat adipocytes and in the mouse eye, respectively. Of this large set of transcription-associated genes, >10% are associated with hormone-mediated transcription. Since it has been previously reported that P2P-R can bind the SRC-1 transcription co-regulatory factor (steroid receptor co-activator 1, [Ncoa1]), the possible effects of P2P-R on estrogen-induced transcription were evaluated. Estrogen-induced transcription was repressed 50-70% by the transient transfection of P2P-R plasmid constructs into four different cell types. In addition, knockdown of P2P-R expression using an antisense oligonucleotide increased estrogen-mediated transcription. Co-immunoprecipitation assays confirmed that P2P-R interacts with SRC-1 and also demonstrated that P2P-R interacts with estrogen receptor α.

**Conclusions:**

The findings presented in this study provide strong support for the value of systems genetics, especially GeneNetwork, in discovering new functions of genes that can be confirmed by molecular analysis. More specifically, these data provide evidence that the expression of P2P-R co-varies in a genetically-defined manner with large transcription networks and that P2P-R can function as a co-repressor of estrogen-dependent transcription.

## Background

Systems genetics is an evolving new speciality that can be used to define biological networks and to predict molecular interactions. It is based on the analysis of transcripts whose expression co-vary within genetic populations, such as the BXD strains of mice and the HXB strains of rats or other genetic reference panels. GeneNetwork http://www.genenetwork.org is an example of a bioinformatics tool that can be used to explore systems genetics data.

The importance of defining biological networks and predicting molecular interactions has been emphasized by several reports [1,2]. Such studies emphasize that when knowledge about DNA variation within populations is interfaced with data on gene expression, protein interactions and DNA-protein binding, biological networks can be constructed that are predictive of the physiological molecular interactions and of disease susceptibility.

In 2005, we published the first report documenting the ability of the systems genetics tool GeneNetwork to predict interactions between molecules that could be then confirmed by molecular analysis [3]. The P2P-R gene, coding for a hnRNP-related protein [4] that binds both the p53 [5] and Rb1 [4] tumor suppressor proteins was used as a test molecule. P2P-R was entered into GeneNetwork to search for a co-variant that was most highly co-expressed in three tissues of the BXD mouse genetic reference panel, ie,, cerebellum, hematopoietic stem cells and whole brain specimens. The outcome of that study was the identification of Pum2 as the gene product that showed a strong positive expression covariance with P2P-R. Pum2 is a member of the highly conserved Puf family of RNA binding proteins that often function as gene-specific translation regulators, through their association with specific elements located in the 3' untranslated region (UTR) of their target mRNAs [6,7]. The 3' UTR of the P2P-R mRNA was found to contain one perfect consensus and two near-perfect consensus Pum2 binding sequences, while pull-down assays combined with reverse transcription and RT-PCR confirmed that Pum2 does indeed bind P2P-R mRNA to modulate its expression [3].

From the time when P2P-R was cloned it has been implicated in multiple cellular processes such as mitosis [8,9], mRNA processing [10] and translation [3]. In addition, recent studies have suggested that P2P-R has a role in ubiquitination and degradation of p53 and YB-1 proteins [11,12], while it has been shown to be highly up-regulated in oesophageal cancer, being a promising target for immunotherapy against the disease [13]. To further investigate the functional potential of P2P-R, we used the GeneNetwork database to evaluate if genetically-defined mechanisms modulate the co-expression of any distinct sets of gene products with P2P-R. An adipocyte genetic reference panel of HXB/BXH recombinant strains of rats and an eye genetic reference panel of BXD recombinant inbred strains of mice have been employed for these studies. These two genetic reference panels were chosen because P2P-R was cloned and characterized using primarily proadipocytes [4] and because the GeneNetwork eye database represents the most genetically diverse BXD reference panel available for analysis.

The outcome of these systems genetics studies implies that P2P-R is an important member of large genetically-defined transcription networks in adipose and eye tissues. That prediction was then confirmed by data showing that P2P-R can function as a co-repressor of estrogen-induced transcription. Immunoprecipitation experiments strengthen such functional studies by confirming that P2P-R interacts with SRC-1 and by demonstrating that P2P-R also interacts with estrogen receptor α (ERα). These data suggest that P2P-R may be a component of large macromolecular complexes that impact steroid-dependent transcription.

## Results

### P2P-R is part of a genetically-defined transcription network in fat cells

Since most studies concerning the cloning and characterization of P2P-R were performed using the 3T3T proadipocyte system [4], we initiated the current studies focusing on the rat fat cell GeneNetwork microarray database to evaluate if genetically-defined mechanisms modulate the co-expression of any distinct sets of transcripts with P2P-R. The results show that in rat fat cells [14], P2P-R is co-expressed with a large set of transcription-associated gene products.

**Figure 1:**
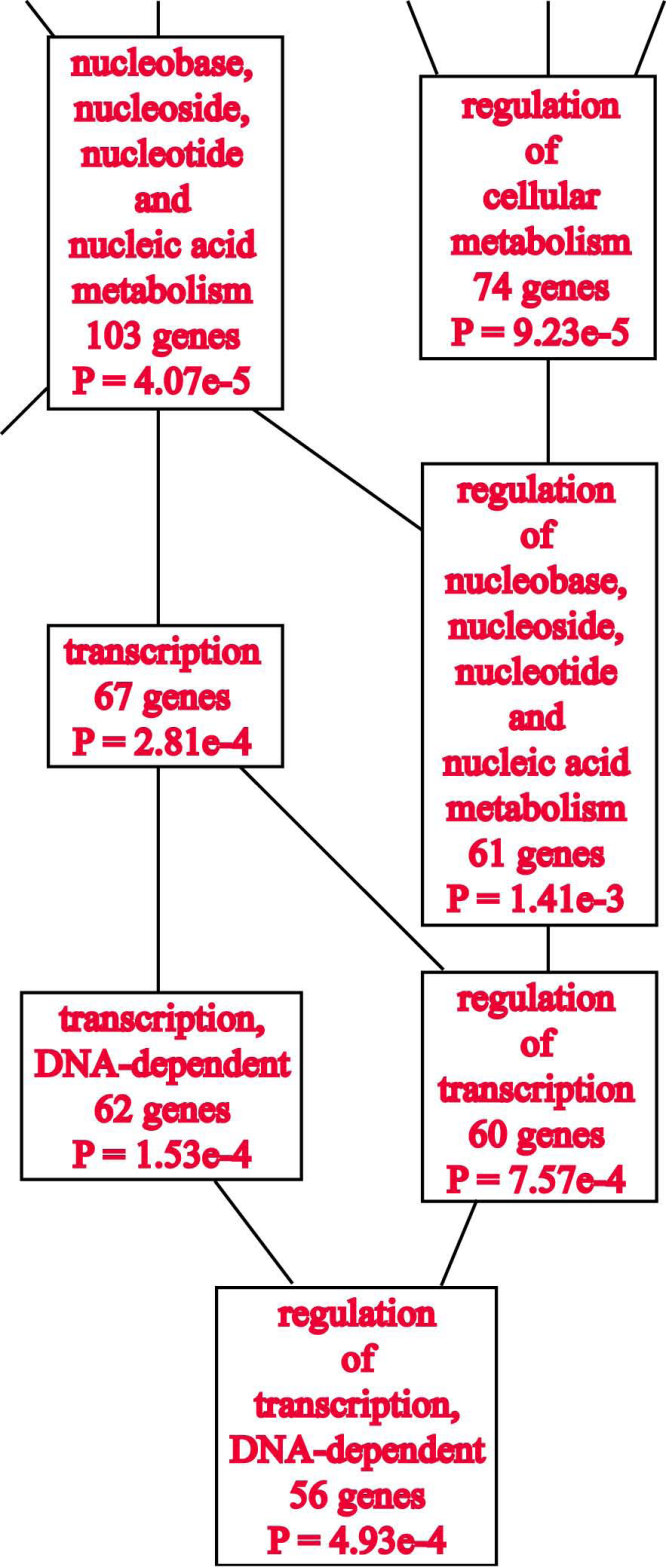
**Discovery of a P2P-R-associated transcription network in fat cells**. The top 1000 gene transcripts whose expression covaries with P2P-R in fat cells derived from the BXH/BHX rat recombinant inbred genetic reference panel were exported from GeneNetwork to the WebGestalt gene ontology analysis system to determine with which functional gene sets P2P-R may be associated. In an excerpt of that report, the data shown document that P2P-R is significantly co-expressed with a set of at least 67 transcription-associated gene products with p = 2.81e-4. Review of the 1000 top covariants with P2P-R using the "Trait Data and Analysis Form - IDs" function of GeneNetwork revealed some required revisions of the WebGestalt data and the addition of a few transcription-associated genes to give a total of 75 transcription genes whose expression co-varies (see Additional file 1).

**Figure 2:**
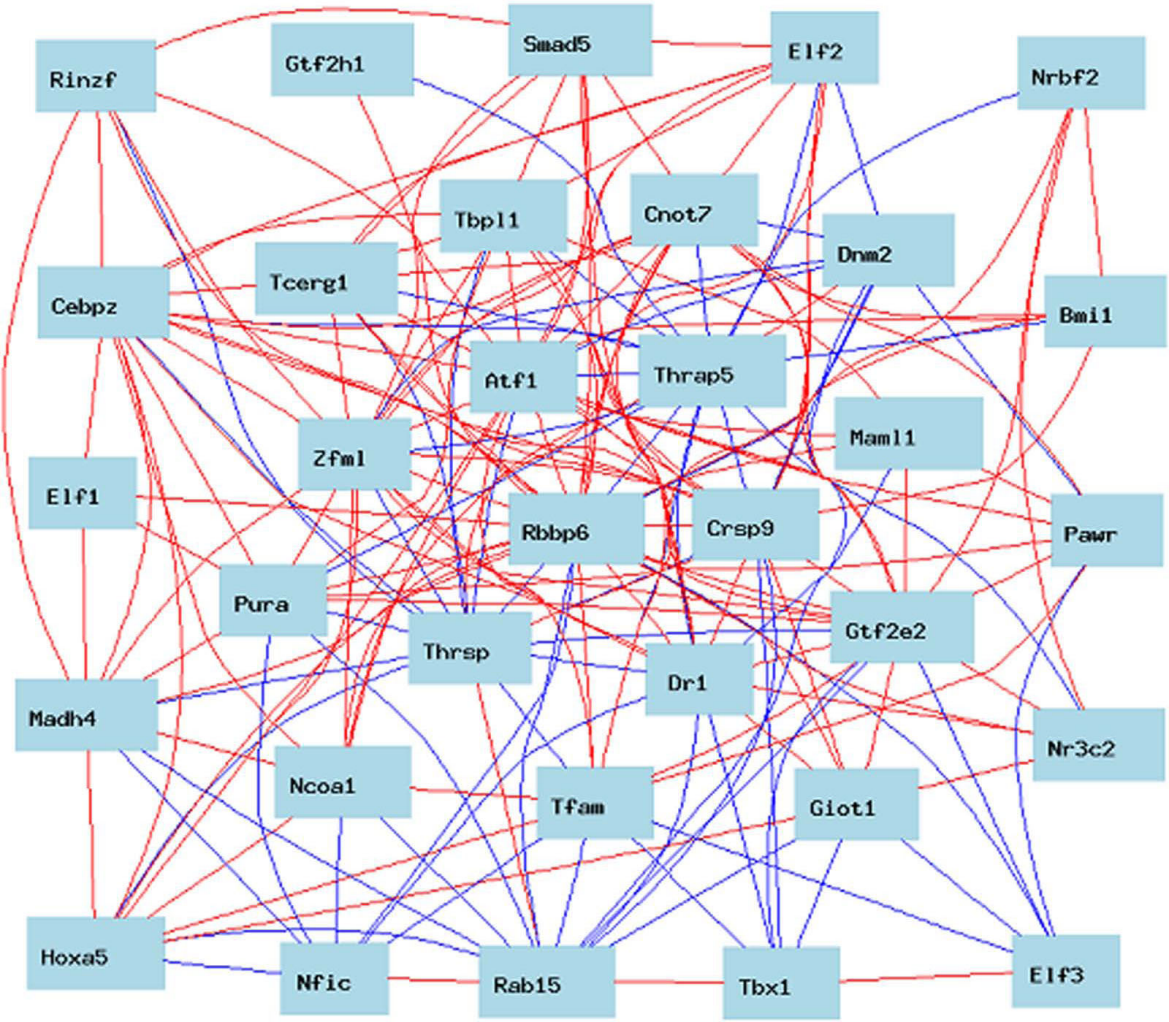
**Characteristics of the P2P-R-associated transcription network in fat cells**. Of the transcription network of 75 gene transcripts in fat cells, the 34 that are co-expressed with a correlation coefficient of ≥ 0.75 are illustrated as a robust transcription network. The color of the lines connecting the 34 gene transcripts indicate the degree to which their expression co-varies among the strains evaluated (red = very high positive correlation, orange = moderate positive correlation, blue = very high negative correlation; green = moderate negative correlation). At the core of this network a group of 10 transcripts that show the most numerous associations is evident. These key transcription network components include: P2P-R (Rbbp6), Atf1, Thrap5, Crsp9, Zfml, Pura, Dr1, Gtf2e2, Maml1 and Cnot7.

When the top 1000 gene transcripts that are co-expressed with P2P-R and have correlation coefficients ≥ 0.67 were exported for gene ontology analysis to WebGestalt, 67 gene products were found to be associated with transcription with p = 2.81e-4 (Figure 1). When these top 1000 transcripts were rechecked, using the Trait Data and Analysis Form - ID function of Gene Network, additional gene products associated with transcription were identified, to give a total of 75 transcription-associated gene products, including P2P-R, whose expression genetically co-varies (Additional file 1) [Alternate designations for some of these transcripts include: Smad7 (Madh7), Yt521 (Ythdc1), Smad4 (Madh4), Jmjd1c (Pr), Taf5 (Usmg5), Garnl1 (Tulip) and Pcgf4 (Bmi4)]. Figure 2 shows that of the transcription network consisting of 75 gene transcripts, 34 are co-expressed with a correlation coefficient of ≥ 0.75. Figure 2 also suggests that at the core of this network is a group of 10 transcripts that show the most numerous genetic associations. These key transcription network components include: P2P-R (Rbbp6), Atf1, Thrap5, Crsp9, Zfml, Pura, Dr1, Gtf2e2, Maml1 and Cnot7.

The above data were then normalized using the Robust Multiarray Method (RMA, http://odin.mdacc.tmc.edu/~zhangli/PerfectMatch/) [15]. In such studies it is worthwhile to evaluate databases using at least two normalization approaches. Therefore, the Mas5 normalization tool was also used. Both normalization methods confirmed that a large network of transcription-association gene transcripts is indeed co-expressed with P2P-R in rat fat cells (data not shown). In support of the above data based on analysis of the fat cell GeneNetwork database derived from HXB/BHX recombinant inbred rats, limited analysis were also performed on the GeneNetwork mouse adipose tissue database wherein it was found that 13.5% of the transcripts whose expression co-varies with P2P-R with a correlation coefficient of ≥ 0.5 has transcription-associated functions (data not shown). This percentage is comparable to the one observed in studies on the fat cell database of recombinant inbred HXB/BHX rats.

### P2P-R is part of a genetically-defined transcription network in the eye

**Figure 3:**
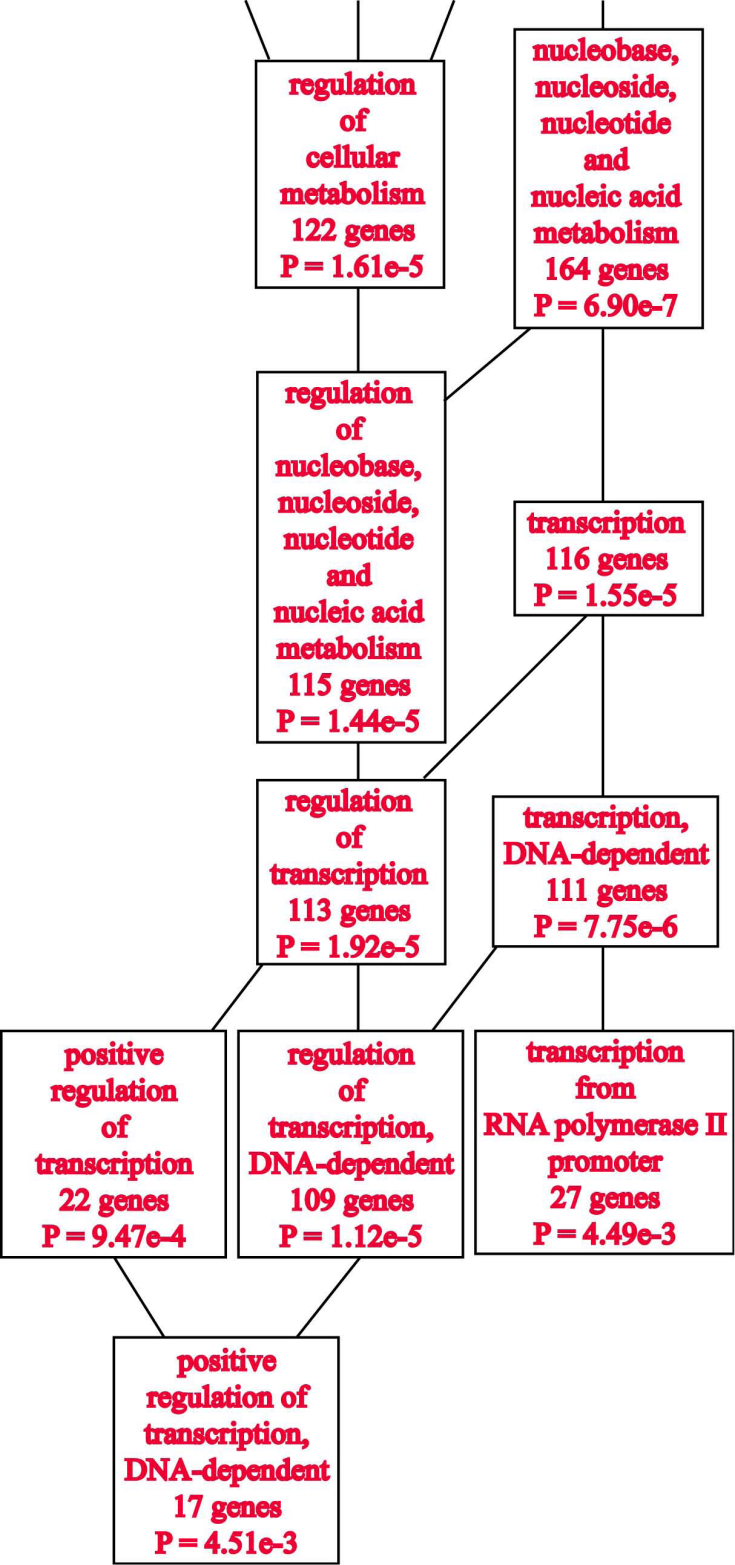
**Discovery of a P2P-R-associated transcription network in eye tissue**. The top 1000 gene transcripts whose expression co-varies with P2PR in the eye tissue derived from the BXD mouse recombinant inbred genetic reference panel was exported from GeneNetwork to the WebGestalt gene ontology analysis system to determine with which functional gene sets P2P-R may be associated. In an excerpt of that report, the date shown documents that P2P-R is significantly co-expressed with a set of at least 116 transcription-associated gene products with p = 1.55e-5. This set of co-expressed gene transcripts had correlation coefficients of ≥ 0.65. Review of the 1000 top covariants with P2P-R using the "Trait Data and Analysis Form - IDs" function of GeneNetwork revealed some required revisions of the WebGestalt data and the addition of a few transcription-associated genes to give a total of 135 transcription genes whose expression co-varies (see Additional file 2).

**Figure 4:**
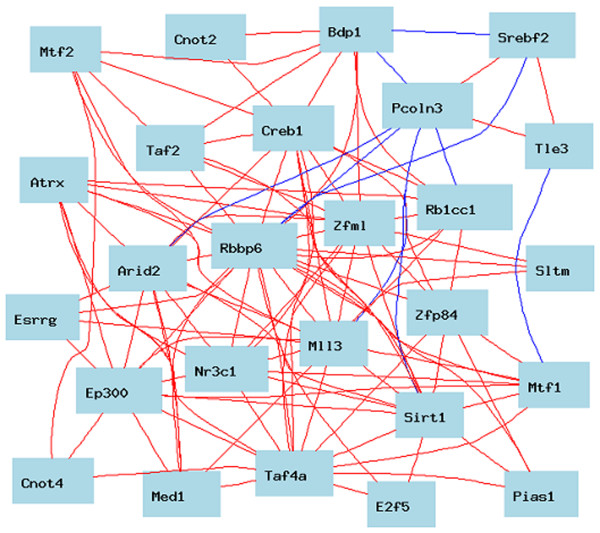
**Characteristics of the P2P-R-associated transcription network in eye tissue**. The transcription network of 26 genes of the BXD eye tissue that are co-expressed with correlation coefficient of ≥ 0.75 are illustrated. The color of the lines connecting the gene products of this transcription network indicate the degree to which their expression co-varies among the strains evaluated (red = very high positive correlation, orange = moderate positive correlation, blue = very high negative correlation; green = moderate negative correlation). It is apparent that P2P-R (Rbbp6) is of central importance in this network.

To confirm the finding that P2P-R is part of a large transcription network, evaluations were next performed in other tissues. In this respect, the eye GeneNetwork database was evaluated because it includes the largest and most genetically diversity mouse population with >100 strains [16]. In the context of this study, the top 1000 gene transcripts whose expression co-varies with P2P-R and had correlation coefficients of ≥ 0.65 were identified and exported to the online WebGestalt gene ontology analysis system. Figure 3 presents an excerpt of that analysis showing that 116 gene products with transcription-associated functions are co-expressed with P2P-R with p = 1.55e-5. When the top 1000 transcripts were manually reappraised as above, a total of 135 transcription gene products were identified as part of the transcription network associated with P2P-R (probeset 1426487). A tabulation of all these transcription-associated gene products are presented in Additional file 2, and Figure 4 illustrates the 26 transcripts of that network whose expression covary with correlation coefficients >0.75.

Even more detailed analysis of the 135 members of the eye transcription network, reveals the following network characteristics:

1) ~80% of the 135 transcription network gene products have nucleotide binding characteristics.

2) >40% of the 135 transcription network gene products bind zinc.

3) ~30% of the 135 transcription network gene products bind both nucleotides and zinc, and

4) ~10% of the 135 members of the P2P-R transcription network gene products are associated with hormone-mediated transcription. These include the following transcription factors or co-factors: Nr3c1 [+0.8472], Ep300 [+0.8056], Pparbp [+0.7709], Glcci1 [+0.7690], Esrrg [+0.7574], Srebf2 [-0.7568], Ncoa6 [+0.7423], Ncor1 [+0.7115], Ncoa1 (Src-1) [+0.6987] and Ncoa2 [+0.6787] and Nr2f6 [-0.6653] (the parenthetic numbers indicate positive or negative expression correlation coefficients with P2P-R expression).

The findings from studies on fat cell-adipose and eye tissues therefore strongly suggest that P2P-R has the potential to function as a member of a genetically-defined transcription complex. Two important questions arise from these findings. Does a P2P-R-associated transcription network exist in all tissues? Can the role of P2P-R in transcription be confirmed by molecular studies? To address the first question, studies were performed on several other tissues in the GeneNetwork database to search for additional P2P-R transcription networks. There was no evidence of a P2P-R-associated transcription network in liver or whole brain databases, however, in a cerebellum database a smaller P2P-R-associated transcription set could be identified (data not shown). These data suggest that the role of P2P-R in transcription may be tissue specific. The studies in the following sections address the second question and show that P2P-R can indeed function as a transcriptional co-repressor.

### Repression of estrogen-induced transcription by P2P-R

Several members of the transcription network co-expressed with P2P-R participate in steroid-regulated transcription (Nr3c1, Glcci1, Esrrg, Srebf2, Ncoa6, Ncor1, Ncoa1, Ncoa2). In this regard, of special note is the April 1, 2005 report in the **Nuclear Receptor Signaling Atlas **online database http://www.nursa.org that P2P-R interacts with Ncoa1 (SRC-1), a well-known steroid receptor co-regulator (for review see ref. [17]. This datum was placed in the NURSA database by Jung SY, Luo H, Malovannaya A, Kim T, Zhang J, Qin J and O'Malley BW under the designation of proteomic analysis of steady-state nuclear hormone receptor coactivator complexes.

**Figure 5:**
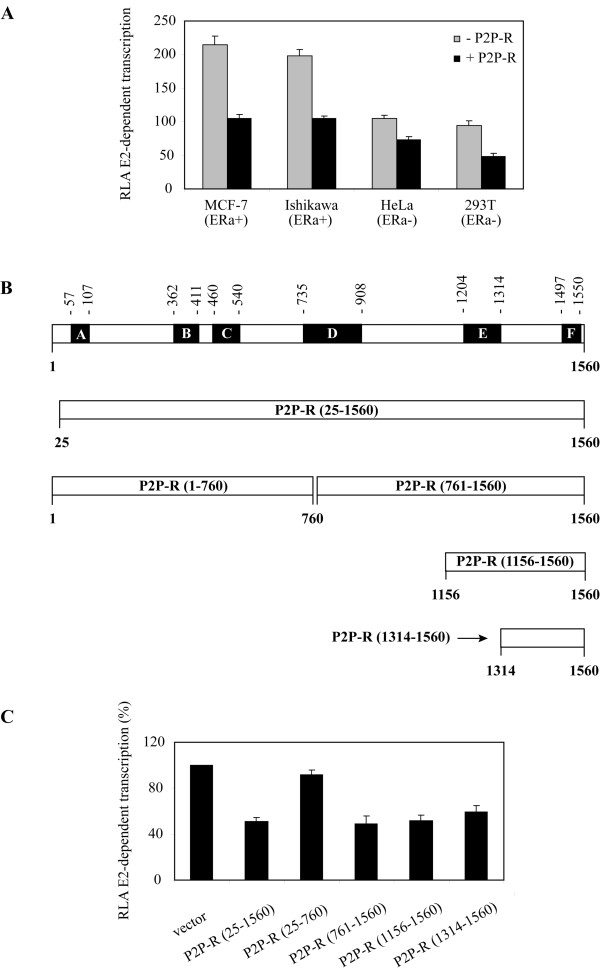
**P2P-R inhibits estrogen-dependent transcription**. (A) Functional assays documenting that the transient transfection of FLAG-tagged P2P-R into MCF-7, Ishikawa, HeLa and 293T cells co-represses estrogen-induced transcription. MCF-7 and Ishikawa cells, that contain endogenous ERα, were transfected with 300 ng of luciferase reporter construct (pGl2-ERE2X-TK) plus renilla luciferase plasmid (15 ng/well), with or without co-transfection with 1 μg of FLAG-tagged P2P-R(25-1560). HeLa and 293T cells, that lack endogenous ERα, were also co-transfected with 50 ng pSG5-ERα. Cells were then incubated for 18 h in phenol-red free DMEM with 5% CS-FBS in the presence of DMSO or 100 pM 17-beta estradiol (E2). Data are expressed as Relative Luciferase Activity (RLA) of E2-dependent transcription (see Methods). Experiments were performed in triplicate, *bars *denote standard error. (B) Characteristic domains of the P2P-R protein (1-1560 aa) and definition of the five P2P-R constructs used in transcription assays. **A**, ring type zinc finger domain, **B**, proline-rich domain, **C**, SR-like domain, **D**, Rb1 binding domain, **E**, p53 binding domain, and **F**, lysine-rich domain. (C) Functional assays measuring the effects of expression of specific P2P-R domains on E2-dependent transcription in MCF-7 cells. Cells were transfected with 300 ng of pGl2-ERE2X-TK plus renilla luciferase plasmid (15 ng/well) and 1 μg of 3 × FLAG-myc-CMV™-29 expressing the respective P2P-R segments and then incubated for 18 h in phenol-red free DMEM with 5% CS-FBS in the presence of DMSO or 100 pM E2. Data are presented as % Relative Luciferase Activity (RLA) of E2-dependent transcription. The value of RLA in the presence of the expression vector only (3 × FLAG-myc CMV™ -29) was set to be 100 (control) so that the data can be presented as % RLA. Standard error determinations were calculated based on three replicate experiments.

Based on these observations, we sought to investigate whether P2P-R impacts estrogen-dependent transcription in cell lines that either contain estrogen receptor α, such as MCF-7 and Ishikawa cells or lack native estrogen receptors, such as HeLa and 293T cells. To this end MCF-7 and Ishikawa cells were transiently co-transfected with an ERα-reporter construct with an empty vector or with a plasmid construct that encodes the near full length P2P-R (aa 25-1560, GenBank database accession no U83913), while HeLa and 293T cells were additionally co-transfected with ERα. Co-transfection experiments were performed in the presence of either vehicle (DMSO) or 100 picomolar 17-beta estradiol (E2) to determine if the transfection of P2P-R influences basal and/or estrogen-dependent transcription. The results presented in Figure 5A document that P2P-R represses E2-induced transcription in all cell lines tested with repression ranging from 49 to 70%. No effect on transcription levels was observed in the presence of DMSO (data not shown), suggesting that P2P-R influences only E2-dependent transcription.

To identify which domain of the P2P-R protein is responsible for the observed repression of E2-induced transcription, MCF-7 cells were transiently co-transfected with an ERα-reporter construct with an empty vector or with plasmid constructs that encode specific P2P-R domains as illustrated in Figure 5B. The five P2P-R plasmid constructs that were employed include: the near full length P2P-R (aa 25-1560); the N-terminal half of P2P-R (aa 1-760); the C-terminal half of P2P-R (aa 761-156); a C-terminal sub-domain of P2P-R (aa 1156-1560) and the C-terminal most domain of P2P-R (aa 1314-1560) [8,18]. As shown in Figure 5C, all four P2P-R constructs that encode the C-terminal regions of P2P-R, repressed estrogen-dependent transcription to more or less the same extent as full-length P2P-R, whereas the N-terminal half of P2P-R showed minimal repressor activity. It appears therefore that a region within the 246 amino acids of the C-terminal most segment of P2P-R is the primary effector that functions to inhibit estrogen-dependent transcription.

**Figure 6:**
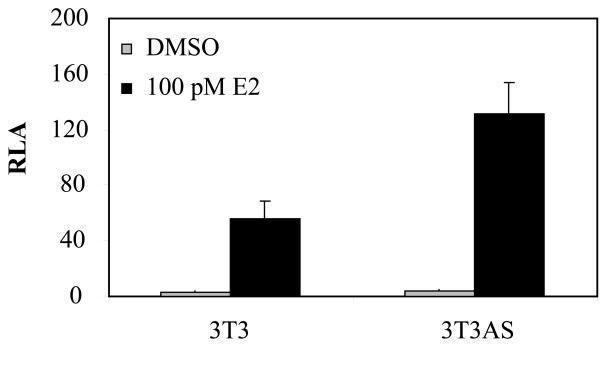
**E2-dependent transcription is increased in 3T3T cells stably transfected with a P2P-R antisense oligonucleotide**. Native 3T3T, and 3T3T cells that have been stably transfected with a P2P-R antisense oligonucleotide (AS), to knockdown P2P-R protein expression, were transfected with ERE-luciferase plasmid (300 ng/well) plus renilla luciferase plasmid (15 ng/well) and pSG5-ERα (50 ng/well) and then incubated for 18-24 h in phenol-red free DMEM with 5% CS-FBS in the presence of DMSO or 100 pM 17 β-estradiol. RLA, relative luciferase activity (RLA represents the firefly luciferase RLU divided by the renilla luciferase RLU for the same sample). Experiments were performed in triplicate, *bars *denote standard error.

To substantiate the conclusion that P2P-R functions as a co-repressor of estrogen-induced transcription, studies were next performed to evaluate the effects of knocking down P2P-R expression on estrogen-dependent transcription. For these studies, mouse 3T3T cells were employed, since a 3T3T cell line that express reduced levels of P2P-R had previously been developed by stably transfecting cells with a construct expressing a P2P-R antisense RNA that knocked down P2P-R expression by 50-70% [9]. The data in Figure 6 demonstrate that 3T3T cells with knocked down P2P-R expression showed a 2.2 fold increase in estrogen-induced transcription activity.

### The P2P-R protein interacts with ERα and SRC-1

**Figure 7:**
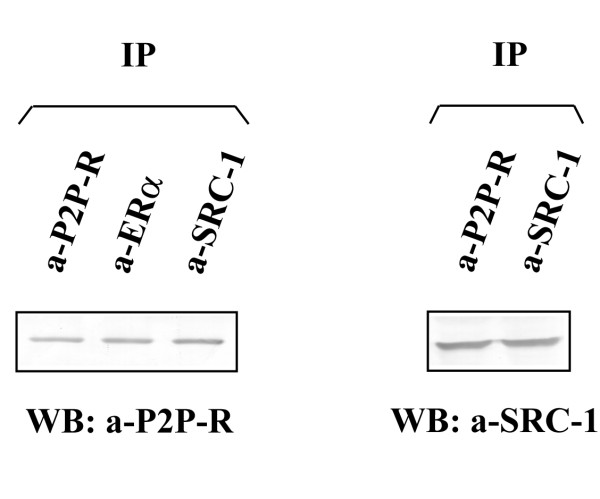
**P2P-R interacts with ERα and SRC-1**. In the left panel lysates were first immunoprecipitated with the anti-P2P-R, the anti-ERα and the anti-SRC-1 antibodies respectively and the immunoprecipitates were subsequently immunoblotted with the anti-P2P-R antibody. In the right panel immunoprecipitations were performed with the anti-P2P-R and the anti-SRC-1 antibodies respectively and the immunoprecipitates were immunoblotted with the anti-SRC-1 antibody. All immunopreciptiation experiments were carried out using HeLa extracts (1 mg/immunopreciptation), whereas MCF-7 cells were used in experiments with ERα. No immunoprecipitation of P2P-R or SRC-1 was observed when an irrelevant anti-GFP monoclonal antibody was used as control for the immunoprecipitation experiments (data not shown).

Since it has been previously reported that P2P-R can interact with steroid receptor coactivator 1 protein (SRC-1) http://www.nursa.org, we sought to investigate the potential interactions of P2P-R with components of the estrogen receptor complex. Accordingly, we evaluated the potential interaction of P2P-R with ERα and attempted to confirm experimentally its interaction with SRC-1. Figure 7A shows that antibodies against ERα and SRC-1 co-immunoprecipitated P2P-R. On the other hand, the anti-P2P-R antibody was able to co-immunoprecipate SRC-1, while no immunoprecipitation of ERα was detected (data not shown). This could be due to the fact that the epitopes recognized by ERα were sterically hindered in the P2P-R complexes. Future experiments will be required to determine if ERα and SRC-1 directly interact with P2P-R and to which P2P-R domains they bind.

## Discussion

The data reported in this paper establish that the function of gene products can be confidently predicted by determining their association with specific genetically-defined biological networks, using systems genetics approaches and tools, such as GeneNetwork. GeneNetwork has been previously used to predict a molecular interaction between P2P-R and Pum2, the gene transcript which is most consistently co-expressed with P2PR in three tissues of the BXD mouse genetic reference panel. That prediction was confirmed by molecular studies showing that Pum2 binds to a specific sequence in the 5'UTR of P2PR mRNA to modulate P2PR expression [3].

The current studies expand the use of GeneNetwork databases in an attempt to identify biological networks in which P2P-R is a member, leading to the prediction of new function(s). Data derived using these systems genetics approaches suggest that P2P-R is an important member of large genetically-defined transcription networks in adipose and eye tissues, thereby having the potential to impact the expression of many hundreds of genes. That prediction was then confirmed by molecular studies showing that P2P-R can indeed function as a transcriptional co-repressor.

The results specifically demonstrate that P2P-R acts as a co-repressor of estrogen-dependent transcription in four different cell systems including MCF-7, Ishakawa, HeLa and 293T cells. The co-repressor activity of P2P-R was further confirmed by evidence that knocking down P2P-R protein expression, using an antisense oligonucleotide, facilitates estrogen-induced transcription. Immunoprecipitation experiments support those functional studies by showing that the P2P-R protein is a component of the estrogen-transcription complex that includes ERα and SRC-1. Preliminary data also suggest that P2P-R interacts with the Mad2 mitotic protein (Scott RE, unpublished observation), which is of interest in this context, since it has previously been reported that Mad2 associates with estrogen receptor beta [19].

An important question concerns how P2P-R might affect estrogen-induced transcription. Since P2P-R is a hnRNP-like protein that associates with the nuclear matrix [4], [Peidis P, Giannakouros T and Scott RE, unpublished observations], we propose that estrogen-dependent transcription might also be linked to the nuclear matrix in association with P2P-R. In this respect it has been reported that estrogen-dependent transcription is mediated by immobilizing the transcription regulatory complex to DNAse resistant nuclear matrix-like entities, in a manner that allows for continued turnover of some of the its components [20]. Furthermore, the scaffold attachment factor B1 (SAFB1) [21], which can bind to the S/MAR (Scaffold/Matrix Attachment Regions) elements of the nuclear matrix, was shown to interact with the C-terminal domain of RNA polymerase II, and with ERα and to suppress ERα-mediated transcription [22-25]. Interestingly, we have previously shown by pull-down assays that the RS domain of P2P-R binds to SAFB1 [26]. We therefore speculate that P2P-R's transcription co-repressor function might stem from its ability to complex with SAFB1 and form dynamic structural and functional entities that influence the organization of chromatin or control the stringency by which various components of the estrogen receptor complex associate with the nuclear matrix, thereby regulating their rate of turnover, and consequently influencing the transcription rate.

It should be also noted that the C-terminal most domain of P2P-R (aa 1314-1560) that is responsible for the observed inhibition of estrogen-dependent transcription is an unusual lysine rich region, reminiscent of the basic N-termini of several histones [4,5]. Core histones are known to undergo acetylation that loosens the DNA-histone contacts, thereby making the DNA more accessible to transcription factors and promoting transcription initiation. The positively charged lysine rich region in P2P-R has been reported to undergo *in vitro *acetylation [5]. It is therefore possible that this region is similarly modified *in vivo*, in a way that mimics histone acetylation, thus acting as a competitive inhibitor of histone acetylation and consequently of chromatin remodeling.

The above suggested mechanisms by which P2P-R might influence transcription will need to be the focus of many more detailed studies. However, based on the data presented in this paper, it is now clear that P2P-R does have an important role in transcription, specifically in estrogen-dependent transcription and that this discovery was predicted using GeneNetwork.

## Conclusions

This study strongly supports the conclusion that GeneNetwork is an excellent tool that can be used to confidently predict new functions for genes. Using GeneNetwork, it was established that large genetically-defined transcription networks that include P2P-R exist in fat cells and eye tissues. Based on a previous report that P2P-R can interact with SRC-1, a well known steroid receptor co-regulator, and given that a significant percentage of the transcription genes within these networks are associated with hormone-mediated transcription, the effects of P2P-R on estrogen-dependent transcription were evaluated. The data presented corroborate the outcome of the systems genetics analyses and clearly show that P2P-R can function as a co-repressor of estrogen-dependent transcription. Immunoprecipitation experiments strengthen those functional studies by confirming that P2P-R interacts with SRC-1 and by demonstrating that P2P-R also interacts with ERα. P2P-R may therefore be a component of large macromolecular complexes that impact steroid-dependent transcription.

## Methods

### Use of the GeneNetwork database to identify genetically-modulated co-expression of P2P-R transcripts with transcription gene products

GeneNetwork is a web resource that contains many large gene expression data sets for populations of rats and mice. Using this system it is possible to extract sets of transcripts that co-vary tightly with a target transcript across genetically diverse populations. Patterns of co-variation can be studied in multiple experimental crosses and in multiple tissues [27,28]. We have used the following subset of the GeneNetwork expression data in the present study:

1. Peritoneal fat cells derived from young males of 32 HXB/BHX recombinant inbred rat strains were the sources of the GeneNetwork database [14]. Microarrays were performed using Affymetrix 230A GeneChip with the RMA2z+8 normalization system. The P2P-R (Rbbp6) probset used was [1376947_at].

2. Eye tissue of pooled female and male specimens derived from 68 recombinant inbred BXD mouse strains plus 35 strains of a mouse diversity panel plus 6 knockout mouse stains were selected to provide maximum diversity to this genetic reference panel [16]. Affymetrix M430 2.0 GeneChips were employed and all data were normalized using the RMA system. The P2P-R (Rbbp6) probeset [1426487_a_at] was primarily used.

Additional details on each of these datasets is available online at http://www.genenetwork.org. In many studies lists of expression covariates were exported to the gene ontology program, WebGestalt, to identify functional gene subsets whose co-expression was enriched [29]. Such enriched sets of genes with common functions are designated genetically-defined networks. When these networks are illustrated in graphic form in various figures of this paper, the color of the lines connecting the genes indicates the degree to which their expression co-varies among the strains evaluated (red = very high positive correlation, orange = moderate positive correlation, blue = very high negative correlation, green = moderate negative correlation).

### Cell lines

Human HeLa, MCF-7, Ishikawa and 293T cells and mouse 3T3T cells were used in this study. All cells were cultured in Dulbecco's modified Eagle's medium supplemented with 10% (v/v) fetal bovine serum (FBS) and antibiotics, unless otherwise described, such as, in assays to measure estrogen-dependent effects where phenol red and charcoal stripped (CS), estrogen-depleted media was employed.

### Steroid-induced Transcription Assays

MCF-7 and Ishikawa cells, that contain endogenous ERα, were cultured in steroid and phenol red free medium for 48 h. The cells were then plated in 12-well plates at 5 × 10^5 ^cells/well in the same medium, allowed to attach overnight and then transfected with 300 ng/well of a luciferase reporter construct (pGl2-ERE2X-TK), containing two copies of the vitellogenin estrogen response element (ERE) linked to the luciferase gene plus 15 ng/well of pRL vector (Promega) expresing renilla luciferase, with or without co-transfection with FLAG-tagged P2P-R constructs that were prepared as previously described [8,18]. HeLa and 293T cells, that lack endogenous ERα, were also co-transfected with plasmid constructs encoding ERα (50 ng pSG5-ERα/well). Transfections were performed using Effectene (Qiagen, Valencia, CA) as per manufacturer's instructions. Five hours after transfection, cultures were refed fresh media with vehicle (DMSO) or with 100 pM 17-beta estradiol and the cells were therein incubated for 18 h at 37°C prior to processing for analysis of the extent of ERE-dependent transcription. Luciferase activity was measured using 30 μl of cell extract and 100 μl of Luciferase Assay Substrate (Promega, Madison, WI) in a Berthold AutoLumat Plus luminometer. The transcription assays were performed in triplicate, as described in the Dual-Luciferase Reporter Assay System Manual (Promega) using *Renilla *Luciferase as internal control. Data are expressed as Relative Luciferase Activity (RLA) of E2-dependent transcription. RLA represents the firefly luciferase RLU (Relative Light Units) divided by those of the renilla luciferase RLU in the presence of E2 minus the firefly luciferase RLU divided by the renilla luciferase RLU in the presence of DMSO. Additional information concerning this method has been previously reported [30].

### Effect of P2P-R knockdown on estrogen-induced transcription

Native 3T3T cells and 3T3T cells that had been stably transfected with a P2P-R anti-sense oligonucleotide resulting in a 50-70% knockdown in P2P-R protein levels [9], were transfected with ERE-luciferase plasmid (300 ng/well) and pSG5-ERα (50 ng/well), using Effectene (Qiagen, Valencia, CA) as per manufacturer instructions. Transfected cells were incubated for 18-24 h in phenol-red free DMEM with 5% CS-FBS in the presence of DMSO or 100 pM 17 β-estradiol. Cells were harvested and luciferase activity was measured.

### Immunoprecipitation and Western blotting

Human HeLa and MCF-7 cells were used to perform co-immunopreciptiation experiments concerning P2P-R, SRC-1 and ERα. These assays employed the following antibodies: anti- P2P-R [#M56 - Santa Cruz Biotechnology, Inc., Santa Cruz, CA], anti-SRC-1 [#05-522 - Upstate Biotechnology, Lake Placid, NY] and anti-ERα [ERα (H-184), #SC-7207, Santa Cruz Biotechnology]. Immunoprecipitations were performed as previously described [8]. Each immunoprecipitation employed approximately one milligram of total cell extract. Native and immunopreciptated proteins were analyzed by Western blotting as previously described [8,18], using the above described antibodies against P2P-R and SRC-1. For Western blotting analysis of ERα, the anti-ERα antibody (D-12) was used [#SC-8005, Santa Cruz Biotechnology].

## Authors' contributions

PP and MEB carried out transcriptional assays and co-immunoprecipitation experiments. RAW participated in GeneNetwork analysis. TG and RES designed the study, supervised data collection and analysis and drafted the manuscript. All authors read and approved the final manuscript.

## Supplementary Material

Additional file 1 **List of fat cell transcription network components**. Seventy five (75) transcription-associated gene products including P2P-R are genetically co-expressed in fat cells derived from the HXB/BXH rat recombinant inbred genetic reference panel. Click here for file

Additional file 2 **List of eye tissue transcription network components**. One hundred thirty five (135) transcription-associate gene products including P2P-R are genetically co-expressed in the eye tissue derived from the BXD mouse recombinant inbred genetic reference panel. Click here for file
