# Supplementary material for: Systems genetics analyses predict a transcription role for P2P-R: Molecular confirmation that P2P-R is a transcriptional co-repressor
Source: BMC Syst Biol. 2010 Feb 25;4:14. doi: 10.1186/1752-0509-4-14 (PMC2843647; doi:10.1186/1752-0509-4-14)
Supplement: Additional file 1 — List of fat cell transcription network components. Seventy five (75) transcription-associated gene products including P2P-R are genetically co-expressed in fat cells derived from the HXB/BXH rat recombinant inbred genetic reference panel. [file 1752-0509-4-14-S1.DOC]

**Table 1.**  **List of fat cell transcription network components.**  Seventy five (75) transcription-associated gene products including P2P-R are genetically co-expressed in fat cells derived from the HXB/BXH rat recombinant inbred genetic reference panel. The probeset numbers of all these transcripts as in GeneNetwork are listed parenthetically.

P2P-R [Rbbp6] (1376947), Atf1 (1389623), Zfml (1373764), Cnot7 (1367515), Thrap5 (1399078/1376528), Tbpl1 (1373242), Cebpz (1373842), Jmjd1c (1388169), Gtf2e2 (1399114), Nrbf2 (1387152), Crsp9 (1377263), Pura (1374170), Bmi1 (1373568), Ncoa1 (1390010), Rinzf (1376125), Nr3c2 (1368476), Giot1 (1368775), Nfic (1371176), Cebpg (1372885), Dnm2 (1369661), Hr (1369336), Pfn1 (1367605), Maml1 (1371878), Tbx1 (1377490), Aebp (1371996), Hoxa5 (1370969), Gtf2h1 (1373094), Ndnl2 (1376704), Thrsp (1371400/1387852/ 1371250), Smad5 (1390373), Elf3 (1374119), Aes (138761), Srebf1 (1371104), Tfam (1367941), Garnl1 (1390217), Trim33 (1389070), Tp53 (1370752), Nedd8 (1398860), Dr1 (1372453), Elf1 (1374137), Smad4 (1386984), Pnrc1 (1370381), Tcerq1(1372151), Gtf3c3 (1372046), Pawr (1368702), Cnot2 (1376067), Yt521 [Ythdc1] (1368063), Nr1h3 (1387365), Smad7 [Madh7] (1368896), Mterf (1387732), Rnf2 (1376805), Strap (1372078), Caskin1 (1368425), Rab15 (1370758), Tgif (1373421), Ccnl1 (1368050), Notch3 (1369329), Ccnh (1368083), Irf2 (1382503), Htr5a (1369463), Klf15 (1368249), Inppl1 (1370651), Hand2 1374451), Taf5 (1376050), Nfia (1369379), Tbx3 (1390627), Csen (1376225), Foxe3 (1370797), Elf2 (1389130), Elf2 (1389130), Ash2l ( 138871), Zfp105 (1399086), Creb1 (1371291), Jmjd1a (1370975), and Cebpd (1387343).
