# Supplementary material for: Systems genetics analyses predict a transcription role for P2P-R: Molecular confirmation that P2P-R is a transcriptional co-repressor
Source: BMC Syst Biol. 2010 Feb 25;4:14. doi: 10.1186/1752-0509-4-14 (PMC2843647; doi:10.1186/1752-0509-4-14)
Supplement: Additional file 2 — List of eye tissue transcription network components. One hundred thirty five (135) transcription-associate gene products including P2P-R are genetically co-expressed in the eye tissue derived from the BXD mouse recombinant inbred genetic reference panel. [file 1752-0509-4-14-S2.DOC]

**Table 2. List of eye tissue transcription network components.** One hundred thirty five (135) transcription-associate gene products including P2P-R are genetically co-expressed in the eye tissue derived from the BXD mouse recombinant inbred genetic reference panel. The probeset numbers of all these transcripts as in GeneNetwork are listed parenthetically.

**Rb1cc1 (1418968), Cops5 (1460171), Ncoa2 (1435233), Zfp451 (1427208), Creb1 (1428755), Hes6 (1452021), Rnf2 (1424873), Atf6 (1435444), Rcor3 ( 1428342), Taf3 (1440187), Jmy (1460577), Bmi1 (1417493), Atf2 (1452116), Mapk8ip (1425679), Pax6 (1425960), Zfp770 (1434682), Mrg1 (1457632), Trp53bp1 (1435609), Cenpb (1426051), Mcm8 (1429557), Plag2 (1417517), Ncoa6 (1423374), Scand1 (1448868), Zfp64 (1438140), Taf4a (1452438), Ss18l1 (1436911), E2f5 (1417444), Phf17 (1426752), Rsrc1 (1448584), Ash1l (1450072), Txnip (1415997), Bcl10 ( 1418972), Fubp1 (1433640), Ncoa6ip (1421904), Ccnc (1417861), Topors (1448838), Zfp37 (1419207), Mtf1 (1428979), Thrap3 (1427408), Dnajc2 (1417657), Esrrg ( 1455267), Mll3 (1434179), Rnf4 (1451072), Lcorl (1455686), Ppargc1a (1434100), Polr2b (1433552), Mtf2 (1449115), Zfp68 (1417549), Wasl (1426776), Cnot4 (1436645), Jmjd1a (1426810), Zfml (1417791),**

**Mad (1422002), Bckdha (1416647), Med25 (1448637), P2P-R [Rbbp6] (1425114),**

**Ing1 (1416860), Rfzank (1419310), Mef2b (1429079), Nr2f6 (1460648), Ctcf (1418330), Nfat5 (1438999), Tcf25 (1424053), Keap1 (1450747), Mll1 (1452377), Npat (1456485), Tle3 (1449554), Pias1 (1455611), Smad3 (1450472), Sltm (1424452), Sirt1 (1418640), Prdm4 (1452208), Tmpo (1426349), Cnot2 (1456576), Stat6 (1421708), Purb (1428254), Zfp62 (1425495), Sqstm1 (1450957), Maml1 (1426769), Zfp2 (1449913), Ncor1 (1423200), Trp53 (1426538), Polr2a (1426242), Tada2l (1452310), Hdac7a (1420812), Carhsp1 (1415976), Hes1 (1418102), Zfp148 (1455483), Mapk14 (1451927), Zfp318 (1430558), Gtf2f1 (1417699), Nr3c1 (1457635/1460303), Tcerg1 (1421033), Zfp91 (1429615), Rfx3 (1437375), Ldb1 (1452024), Bcor (1429438), Zbtb33 (1434022), Phf6 (1454625), Htatsf1 (1454760), Zfx (1425972), Atrx (1433537), Ing3 (1460082), Suhw4 (1426556), Zfp2 (1449913), Kcnh2 (1449544), Rps6ka5 (1431050), Cutl1 (1425611), Sp2 (1426237), Pparbp (1448708), Stat5b (1422102), Ubtf (1460304), Foxk2 (1428354), Ncoa1 (1434515), Pnn (1423324), Hdac9 (1422156), Gtf2a1 (1433511), Rcor1 (1454932), Zfp187 (1457285), Zfp192 (1455778), Sox4 (1419155), Id4 (1423260), Zfp455 (1427217), Homez (14346636), Klf12 (1455521), Taf2 (1434238), Ep300 (1434765), Srebf2 (1426744), Yaf2 (1429839), Arid2 (1454990), Gpbp1 (1423773), Mtf2 (1418515), Bdp1 (1434284), Pcoln3 (1451098), and Zpf84 (1435916).**
